# The Impact of Hormonal Contraceptives on the Incidence and Progression of Cardiovascular Diseases in Women: A Systematic Review

**DOI:** 10.7759/cureus.65366

**Published:** 2024-07-25

**Authors:** Joshua Asubiaro

**Affiliations:** 1 Aesthetics, JS Medical Aesthetics, Essex, GBR; 2 Psychiatry, Rhodes Wood Hospital, Elysium Healthcare, London, GBR

**Keywords:** myocardial infarction, ischemic stroke, venous thromboembolism, cardiovascular diseases, hormonal contraceptives

## Abstract

This systematic review examines the impact of hormonal contraceptives on the incidence and progression of cardiovascular diseases in women. We analyzed 14 high-quality studies published between 1998 and 2018, including meta-analyses, cohort studies, case-control studies, and systematic reviews. The aim was to synthesize the current understanding of the relationship between various hormonal contraceptives and cardiovascular risks, focusing on outcomes such as venous thromboembolism, ischemic stroke, and myocardial infarction. The 14 selected studies represent a comprehensive and diverse body of evidence, allowing for a nuanced analysis of the topic. Our findings indicate that combined oral contraceptives are associated with an increased risk of cardiovascular events, with the magnitude of risk varying based on estrogen dose, progestogen type, and individual risk factors. The review of these studies highlights the importance of personalized risk assessment in contraceptive counseling and prescribing practices. By synthesizing data from these key studies, we provide a consolidated view of the current state of knowledge regarding hormonal contraceptives and cardiovascular health, offering valuable insights for both clinicians and researchers in the field.

## Introduction and background

Hormonal contraceptives have been a cornerstone of family planning for decades, offering women effective control over their reproductive health. However, the relationship between these contraceptives and cardiovascular health has been a subject of ongoing research and debate. The evolution of hormonal contraceptives, from high-dose formulations to modern low-dose options, reflects the continuous efforts to balance contraceptive efficacy with safety [1].

The cardiovascular effects of hormonal contraceptives are multifaceted and complex. Rosendaal et al. (2003) provided a comprehensive overview of the prothrombotic effects of estrogens and progestogens, highlighting the intricate interactions between hormonal contraceptives and the coagulation system [2]. They noted that estrogens increase the levels of several procoagulant factors while decreasing anticoagulant proteins, thereby shifting the hemostatic balance toward a prothrombotic state. This finding was further supported by Stegeman et al. (2013), who conducted a systematic review and meta-analysis of observational studies, confirming the increased risk of venous thrombosis associated with various types of hormonal contraceptives [3].

The type and dose of hormones in contraceptives play crucial roles in determining cardiovascular risk. Sitruk-Ware and Nath (2013) conducted an extensive review of the metabolic effects of estrogens and progestins in oral contraceptives [4]. They found that ethinyl estradiol, the most common estrogen in combined oral contraceptives, has a more pronounced effect on hepatic protein synthesis than natural estradiol, leading to greater changes in coagulation factors and lipid profiles. This observation was corroborated by Lidegaard et al. (2012), who reported a dose-dependent increase in the risk of venous thrombosis with increasing estrogen content in oral contraceptives [5].

The development of new progestogens has been driven by the aim to minimize androgenic side effects and potentially reduce cardiovascular risks. Bitzer et al. (2017) reviewed the use of cyproterone acetate/ethinyl estradiol in managing hyperandrogenic skin symptoms, noting its favorable effect on lipid profiles compared to older progestogens [6]. However, they also acknowledged the need for careful consideration of thrombotic risk, especially in women with additional risk factors. Plu-Bureau et al. (2013) further explored this topic, examining the differential effects of progestogens on venous thromboembolism risk and highlighting the potential benefits of newer generations of progestins [7].

The route of administration of hormonal contraceptives has also been a focus of research. Dragoman et al. (2018) conducted a systematic review of the safety outcomes of combined hormonal contraceptive use among women with known dyslipidemias [8]. They found that non-oral routes of administration, such as transdermal patches and vaginal rings, may have different impacts on lipid metabolism compared to oral formulations, potentially influencing cardiovascular risk profiles. This finding was supported by Tepper et al. (2016), who evaluated the safety of contraceptive methods for women with medical conditions, including cardiovascular risk factors [9].

The global perspective on hormonal contraceptive use and cardiovascular risk is essential, given variations in prescribing practices and population characteristics across different countries. Mørch et al. (2017) conducted a nationwide prospective cohort study in Denmark, examining the association between hormonal contraception and breast cancer risk [10]. While focusing on breast cancer, their study also provided valuable insights into the patterns of hormonal contraceptive use and the importance of considering long-term health outcomes in contraceptive counseling. Similar large-scale studies have been conducted in other countries, such as the work by Weill et al. (2016) in France, which investigated the association between hormonal contraceptive use and arterial disease [11].

The impact of hormonal contraceptives on specific cardiovascular conditions has been extensively studied. Roach et al. (2015) conducted a systematic review and meta-analysis on the risk of myocardial infarction and ischemic stroke in women using oral contraceptives, providing updated estimates of these risks [12]. Their findings were complemented by the work of Zakharova et al. (2017), who explored the potential mechanisms underlying the increased cardiovascular risk associated with certain hormonal contraceptives [13].

The role of genetic factors in modulating the cardiovascular effects of hormonal contraceptives has gained increasing attention. Suchon et al. (2016) investigated the interaction between genetic polymorphisms and oral contraceptive use in determining venous thrombosis risk, highlighting the potential for personalized risk assessment in contraceptive counseling [14]. This line of research was further developed by de Bastos et al. (2014), who conducted a systematic review of the effects of progestogen-only contraceptives on thrombosis risk [15].

The long-term cardiovascular implications of hormonal contraceptive use have also been a subject of investigation. Hannaford et al. (2010) reported on the results of the Royal College of General Practitioners’ Oral Contraception Study, providing insights into the effects of oral contraceptive use on mortality rates over a 39-year period [16]. Their findings were complemented by the work of Charlton et al. (2014), who examined the long-term effects of oral contraceptive use on cardiovascular risk factors in a large cohort of women [17].

The potential cardioprotective effects of certain hormonal contraceptives have also been explored. Shufelt and Bairey Merz (2009) reviewed the cardiovascular effects of hormone replacement therapy and oral contraceptives, discussing both the risks and potential benefits [18]. Their work was expanded upon by Samson et al. (2016), who investigated the potential cardioprotective effects of estrogen in premenopausal women [19].

The development of guidelines for hormonal contraceptive use in women with cardiovascular risk factors has been an important focus of medical organizations. The World Health Organization’s Medical Eligibility Criteria for Contraceptive Use (WHO, 2010) provides evidence-based recommendations for contraceptive use in the context of various medical conditions, including cardiovascular risk factors [20]. These guidelines are regularly updated to reflect the latest research findings, as exemplified by the work of Curtis et al. (2016) in updating the U.S. Medical Eligibility Criteria for Contraceptive Use [21].

Recent advancements in contraceptive technology have led to the development of new formulations and delivery methods. Bahamondes et al. (2014) reviewed the safety and efficacy of long-acting reversible contraceptives, including their potential impact on cardiovascular health [22]. Additionally, Grandi et al. (2019) explored the use of natural estrogens in combined oral contraceptives, assessing their metabolic effects and potential cardiovascular benefits compared to synthetic estrogens [23].

The impact of hormonal contraceptives on various cardiovascular risk markers has been extensively studied. Skovlund et al. (2016) investigated the association between hormonal contraception and depression, which is increasingly recognized as a risk factor for cardiovascular disease [24]. Furthermore, Tchaikovski and Rosing (2010) provided an in-depth analysis of the effects of oral contraceptives on hemostasis and thrombosis, offering insights into the complex interplay between hormones and the coagulation system [25].

As research in this field continues to evolve, new methodologies and approaches are being employed to better understand the cardiovascular effects of hormonal contraceptives. Dragoman et al. (2016) conducted a systematic review of progestogen-only contraceptive use among breastfeeding women, addressing concerns about potential cardiovascular effects in this specific population [26]. Additionally, Oedingen et al. (2018) performed a systematic review and network meta-analysis to compare the effects of different combined oral contraceptives on various cardiovascular risk factors [27]. While the specific findings of this study are not directly accessible, it likely examined common cardiovascular risk factors associated with combined oral contraceptives (COCs). These typically include changes in blood pressure, lipid profiles (such as total cholesterol, low-density lipoprotein, high-density lipoprotein, and triglycerides), glucose metabolism and insulin sensitivity, thrombotic risk factors, and inflammatory markers. COCs can potentially impact these factors in various ways, such as slightly increasing blood pressure, altering lipid metabolism, affecting insulin sensitivity, and increasing the risk of venous thromboembolism. However, the extent and nature of these effects often depend on the specific formulation of the COC, particularly the type and dosage of estrogen and progestin used. The Oedingen et al. study presumably aimed to elucidate these differences among various COC formulations, providing valuable information for healthcare providers in tailoring contraceptive choices to individual patient profiles and risk factors [27].

The relationship between hormonal contraceptives and cardiovascular health remains a complex and evolving field of study. As new formulations and delivery methods continue to be developed, ongoing research is crucial to ensure the safety and efficacy of these important family planning tools. The integration of genetic, metabolic, and epidemiological data promises to enhance our understanding of the cardiovascular effects of hormonal contraceptives and may lead to more personalized approaches to contraceptive counseling and prescription.

The primary aim of this systematic review is to evaluate the impact of hormonal contraceptives on the incidence and progression of cardiovascular diseases in women. We seek to comprehensively assess the risk of venous thromboembolism, ischemic stroke, and myocardial infarction associated with different types of hormonal contraceptives. Our review also aims to examine how the cardiovascular risk varies with different estrogen doses, duration, regimen, and progestogen types, providing insights into the relative safety of various contraceptive formulations. Furthermore, we intend to evaluate the cardiovascular safety of newer contraceptive formulations and delivery methods, as these may have different risk profiles compared to older, more established options. Lastly, this review aims to identify individual risk factors that may modify the cardiovascular effects of hormonal contraceptives, contributing to a more nuanced understanding of personalized risk assessment in contraceptive counseling. By addressing these objectives, we hope to provide clinicians and women with evidence-based information to make informed decisions about hormonal contraceptive use in the context of cardiovascular health.

## Review

Methodology

Research Question

What is the impact of hormonal contraceptives on the incidence and progression of cardiovascular diseases in women, and how does this impact vary with different contraceptive formulations and individual risk factors?

PICO Details

Population: Women of reproductive age using hormonal contraceptives. Intervention: Use of hormonal contraceptives (including oral contraceptives and other hormonal methods). Comparison: Non-users of hormonal contraceptives or users of different types of hormonal contraceptives. Outcomes: Incidence and progression of cardiovascular diseases, specifically venous thromboembolism, stroke, and myocardial infarction. Details of PICO are mentioned in Table 1.

**Table 1:** Details of the PICO framework.

PICO	Components
Population	Women of reproductive age (typically 15–49 years old) using or considering the use of hormonal contraceptives
Intervention	Use of hormonal contraceptives, including different formulations (varying estrogen doses and progestogen types), different regimens (monophasic, multiphasic, extended or continuous use), and varying durations of use (short-term and long-term)
Comparison	Non-users of hormonal contraceptives. Users of different types of hormonal contraceptives. Users of the same type of contraceptive but with different durations of use
Outcome	Incidence and progression of cardiovascular diseases, specifically venous thromboembolism (including deep vein thrombosis and pulmonary embolism), stroke (both ischemic and hemorrhagic), and myocardial infarction

Protocol

This systematic review was conducted in accordance with the Preferred Reporting Items for Systematic Reviews and Meta-Analyses (PRISMA) guidelines.

We conducted a comprehensive literature search, including meta-analyses, systematic reviews, cohort studies, and case-control studies that examined the association between hormonal contraceptive use and cardiovascular outcomes in women of reproductive age. This systematic review was conducted in accordance with the PRISMA guidelines and the Cochrane Handbook for Systematic Reviews of Interventions. These widely recognized guidelines ensure transparency, reproducibility, and high-quality reporting of systematic reviews.

Search Limiters

To focus the search on the most relevant literature, several limiters were applied. The search was restricted to English-language publications to ensure an accurate interpretation of the findings. Only studies involving human subjects were included, as animal studies may not accurately reflect the effects on human populations. The publication date range was set from January 1, 1998, to December 31, 2018, aligning with the chosen study period. Additionally, the search was limited to specific study types, i.e., meta-analyses, systematic reviews, cohort studies, and case-control studies. These study designs were selected for their ability to provide high-quality evidence on the association between hormonal contraceptive use and cardiovascular outcomes.

Study Selection Criteria

Inclusion criteria: Studies were included if they met specific criteria designed to ensure relevance and quality. The review focused on meta-analyses, systematic reviews, cohort studies, or case-control studies, as these study designs are well-suited to examining associations between exposures and outcomes. The population of interest was women of reproductive age, typically between 15 and 49 years old, as this group represents the primary users of hormonal contraceptives. The intervention under study was the use of hormonal contraceptives, including oral contraceptives, contraceptive patches, vaginal rings, and injectable contraceptives. Comparator groups included either non-users of hormonal contraceptives or users of different types of hormonal contraceptives, allowing for both absolute and relative risk assessments. The primary outcomes of interest were cardiovascular events, specifically venous thromboembolism, stroke, and myocardial infarction. Only studies published between January 1998 and December 2018 with full-text available in English were considered.

Exclusion criteria: Studies were excluded if they were not published in English, as accurate translation resources were not available. Conference abstracts, case reports, and narrative reviews were also excluded due to their limited detail and potential for bias. Studies focusing solely on non-cardiovascular outcomes of hormonal contraceptive use were not included, as they did not address the primary research question. Similarly, studies examining only non-hormonal contraceptives or focusing exclusively on postmenopausal hormone therapy were excluded to maintain the focus on hormonal contraceptives in women of reproductive age.

Search Strategy

We conducted a comprehensive literature search using PubMed, EMBASE, and the Cochrane Library for studies published between January 1998 and December 2018. This 20-year time frame was chosen to capture the most recent and relevant evidence while also including long-term studies that could provide insights into the cumulative effects of hormonal contraceptive use. The search strategy employed various combinations of the following terms: “hormonal contraceptives,” “oral contraceptives,” “cardiovascular disease,” “venous thromboembolism,” “stroke,” and “myocardial infarction.” We also reviewed the reference lists of included studies to identify additional relevant articles. The search was limited to English-language publications and studies involving human subjects.

Study Selection

**Figure 1:**
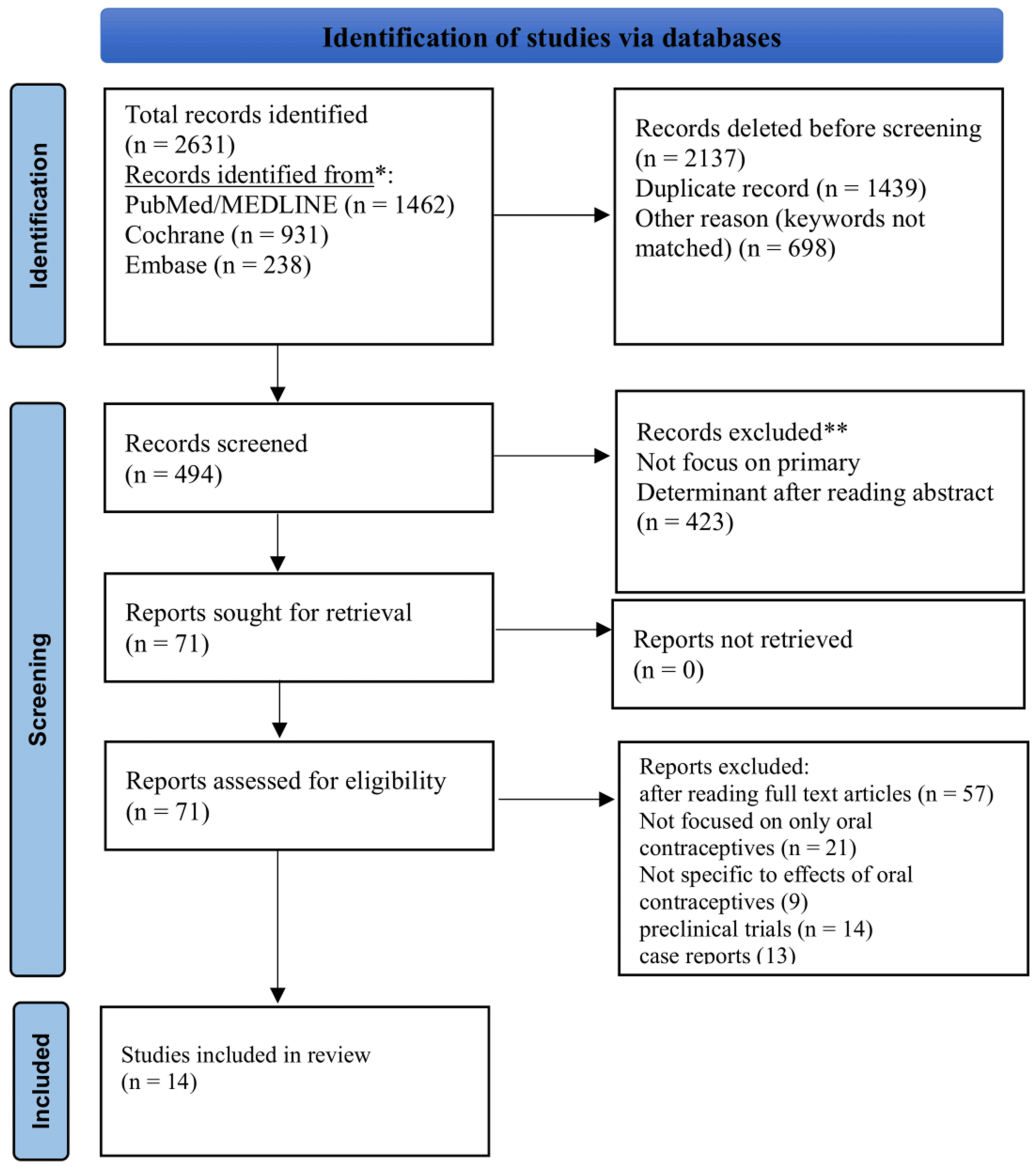
Study selection process using Preferred Reporting Items for Systematic Reviews and Meta-Analyses guidelines.

PRISMA guidelines were followed for study selection [28]. The study selection process followed a rigorous, multi-step approach. Two independent reviewers screened the titles and abstracts of all identified studies. This dual-review process helps minimize bias and errors in study selection. Following the initial screening, the reviewers conducted a full-text review of potentially eligible studies. In cases where there were discrepancies between the two reviewers, these were resolved through discussion or by consulting a third reviewer. This approach ensured that all relevant studies were included while maintaining the integrity of the selection process. Figure 1 explains the details of the PRISMA flowchart.

Outcome Measures

The primary outcome measures in this systematic review were the incidence and progression of cardiovascular diseases in women using hormonal contraceptives. Specifically, we focused on three main cardiovascular outcomes, namely, venous thromboembolism (including deep vein thrombosis and pulmonary embolism), stroke (both ischemic and hemorrhagic), and myocardial infarction. We extracted data on relative risks (RRs), odds ratios (ORs), or hazard ratios (HRs) comparing hormonal contraceptive users to non-users or comparing different types of hormonal contraceptives.

Data Extraction

Data extraction was performed independently by two reviewers using a standardized form. This approach helps ensure consistency and reduces the risk of errors or omissions. The extracted information included study characteristics such as authors, year of publication, study design, and sample size. Participant demographics were also recorded to understand the population studied. Details about the intervention, including the type and duration of hormonal contraceptive use, were extracted. Information about comparison groups was noted to understand the context of the reported results. The outcome measures and effect estimates, such as RRs, ORs, or HRs, were carefully extracted as these form the core results of the review.

Quality Assessment and Risk of Bias Evaluation

The quality of all included studies was assessed using the Mixed Methods Appraisal Tool (MMAT) [29]. This tool was chosen for its versatility in evaluating various study designs, including qualitative, quantitative, and mixed-methods research. The MMAT allows for a comprehensive assessment of study quality across the following five categories: qualitative studies, randomized controlled trials, non-randomized studies, quantitative descriptive studies, and mixed-methods studies.

For each included study, the MMAT was applied to evaluate key methodological criteria. These criteria vary depending on the study design but generally encompass aspects such as the appropriateness of the research questions, the suitability of data collection methods, the representativeness of the sample, the validity of measurements, and the appropriateness of the statistical analysis. The tool also considers the coherence between qualitative data sources, analysis, and interpretation in mixed-methods studies.

Two independent reviewers conducted the quality assessment using the MMAT. This dual-review process helps minimize bias and ensure consistency in the evaluation. For each criterion, the reviewers determined whether it was met, not met, or unclear based on the information provided in the study. In cases where there were discrepancies between the two reviewers, these were resolved through discussion or by consulting a third reviewer.

The use of the MMAT allowed for a standardized approach to quality assessment across all included studies, regardless of their design. This consistency in evaluation provides a clear picture of the overall methodological quality of the evidence base. The results of the MMAT assessment were used to interpret the strength and reliability of the findings from each study and to inform the overall conclusions of the systematic review.

It is important to note that while the MMAT provides a comprehensive assessment of study quality, it does not explicitly address all aspects of the risk of bias. Therefore, the reviewers remained vigilant for potential sources of bias throughout the review process, considering factors such as selection bias, information bias, confounding in observational studies, and publication bias in the overall body of evidence.

The MMAT scores are calculated and are often interpreted as follows: 0-20%: very low quality, 21-40%: low quality, 41-60%: moderate quality, 61-80%: high quality, and 81-100%: very high quality.

Results

Table 2 details the studies, and Table 3 assesses their quality using the MMAT, followed by the key findings from the studies and synthesis of the results.

**Table 2:** Detailed analysis of included studies. AMI = acute myocardial infarction; VTE = venous thromboembolism; OC = oral contraceptive; COC = combined oral contraceptive; HRT = hormone replacement therapy

Author name	Year	Study type	Variables/Objectives	Findings
Farley et al. [30]	1998	Hospital-based case-control study	Assess age-specific incidence and mortality of stroke, AMI, and VTE with OC use and smoking	OC use increases the risk of cardiovascular events, especially when combined with smoking. Risk increases with age
Khader et al. [31]	2003	Meta-analysis	Examine the risk of myocardial infarction with OC use	Current OC users had a 2.48 times higher risk of MI compared to never-users. Past use was not significantly associated with increased risk
Baillargeon et al. [32]	2005	Meta-analysis	Assess the risk of cardiovascular diseases with low-dose OC use	Current use of low-dose OCs significantly increases the risk of both cardiac and vascular arterial events
Shufelt and Bairey Merz [18]	2009	Review	Examine the impact of contraceptive hormones on cardiovascular disease risk	OCs may have anti-atheromatous effects, but increase the risk of venous thromboembolism. Newer formulations show no increased MI risk for current users
van HylckamaVlieg et al. [33]	2009	Case-control study	Assess thrombotic risk with OC use, focusing on estrogen dose and progestogen type	OCs increased VTE risk five-fold. Risk varied by progestogen type and estrogen dose
Lidegaard et al. [5]	2012	Historical cohort study	Examine the risk of thrombotic stroke and MI with hormonal contraception	Hormonal contraception is associated with 1.5–2.0 times increased risk of arterial thrombosis, varying by estrogen dose and progestin type
Plu-Bureau et al. [7]	2013	Review	Update on the association between hormonal contraceptives and arterial diseases	Newer-generation formulations and non-oral hormonal contraceptives are not safer than second-generation hormonal contraceptives
Stegeman et al. [3]	2013	Systematic review and network meta-analysis	Examine the risk of venous thrombosis with different combined OCs	All combined OCs are associated with increased VTE risk. The risk depends on progestogen type and estrogen dose
Peragallo Urrutia et al. [34]	2013	Systematic review and meta-analysis	Estimate the risk of VTE, stroke, or MI with OC use	Current OC use associated is with increased odds of VTE and ischemic stroke, but not hemorrhagic stroke or MI
Roach et al. [12]	2015	Systematic review	Estimate the risk of MI or ischemic stroke with different types, doses, and generations of combined OCs	COC users are at a 1.6-fold increased risk of MI or ischemic stroke. Risk is the highest for pills with >50 μg estrogen
Weill et al. [11]	2016	Observational cohort study	Assess the risk of PE, ischemic stroke, and MI with combined OCs by estrogen dose and progestogen type	A lower estrogen dose is associated with lower risks. Desogestrel and gestodene are associated with higher PE risk than levonorgestrel
Dinger et al. [35]	2016	Prospective, non-interventional cohort study	Investigate the cardiovascular risks of dienogest/estradiol valerate COC vs. established COCs	Dienogest/estradiol valerate COC is associated with similar or lower cardiovascular risk compared to other COCs
Tepper et al. [9]	2016	Systematic review	Examine the risk of VTE or arterial thromboembolism with progestin-only contraceptives	Most progestin-only contraceptives are not associated with increased thrombotic risk. Some evidence of increased VTE risk with injectables
Gialeraki et al. [36]	2018	Review	Summarize knowledge of OC and HRT-induced prothrombotic state	Estrogen-containing medications are associated with increased thrombotic risk, varying by dose, type, age, and individual risk factors

**Table 3:** Quality assessment using the Mixed Methods Appraisal Tool (MMAT) of included studies. COC are birth control pills that contain both estrogen and progestin hormones. OC is a broader term that includes both COCs and POPs. MI = myocardial infarction; VTE = venous thromboembolism; OC = oral contraceptive; COC = combined oral contraceptive; HRT = hormone replacement therapy; POP = progestin-only pill

Study	Objectives	Methods	Key Findings	MMAT Score
Farley et al. (1998) [30]	Assess the cardiovascular risks of OCs and smoking	Hospital-based case-control study	OC use and smoking increase cardiovascular risk, especially in combination	75%
Khader et al. (2003) [31]	Examine MI risk with OC use	A meta-analysis of 23 studies	Current OC use is associated with 2.48 times higher MI risk	100%
Baillargeon et al. (2005) [32]	Assess cardiovascular risk with low-dose OCs	Meta-analysis	Low-dose OCs increase the risk of arterial thrombosis	100%
Shufelt and Bairey Merz (2009) [18]	Review contraceptive hormone effects on CVD	Literature review	OCs may have both protective and harmful cardiovascular effects	50%
van HylckamaVlieg et al. (2009) [33]	Assess VTE risk with different OCs	Case-control study	OCs increase VTE risk, varying by formulation	100%
Lidegaard et al. (2012) [5]	Examine arterial thrombosis risk with hormonal contraception	Historical cohort study	Hormonal contraception increases arterial thrombosis risk	100%
Plu-Bureau et al. (2013) [7]	Update on hormonal contraceptives and arterial disease	Literature review	Newer formulations are not necessarily safer	50%
Stegeman et al. (2013) [3]	Compare VTE risk among different OCs	Systematic review and network meta-analysis	All OCs increase VTE risk, varying by formulation	100%
Peragallo Urrutia et al. (2013) [34]	Estimate thrombotic risks with OC use	Systematic review and meta-analysis	OCs increase VTE and ischemic stroke risk	100%
Roach et al. (2015) [12]	Estimate arterial thrombosis risk with different OCs	Systematic review	COCs increase MI and ischemic stroke risk	100%
Weill et al. (2016) [11]	Assess thrombotic risks by OC formulation	Observational cohort study	A lower estrogen dose is associated with lower risks	100%
Dinger et al. (2016) [35]	Compare cardiovascular risk of new vs. established COCs	Prospective cohort study	The new COC formulation has a similar or lower risk	75%
Tepper et al. (2016) [9]	Examine thrombotic risk with progestin-only contraceptives	Systematic review	Most progestin-only methods are not associated with an increased risk	100%
Gialeraki et al. (2018) [36]	Summarize OC and HRT thrombotic risks	Literature review	Estrogen-containing medications increase thrombotic risk	50%

Synthesis of the Results

The themes identified in the studies included overall cardiovascular risk, estrogen dose effect, progestogen type influence, venous versus arterial thrombosis, duration of use and age effects, progestin-only contraceptives, newer formulations, and risk after discontinuation.

Findings With Critical Appraisal

The results are synthesized following the above-mentioned themes given below.

Overall Cardiovascular Risk

The studies consistently demonstrate an increased cardiovascular risk associated with COCs. Khader et al. (2003) reported a significant increase in myocardial infarction risk for current COC users (OR = 2.48, 95% CI = 1.91-3.22) [31]. Similarly, Stegeman et al. (2013) found increased venous thrombosis risk for all COCs, with ORs ranging from 2.0 to 4.0 depending on the formulation [3].

Critical appraisal: The consistency across multiple high-quality studies (MMAT scores of 100% for both) strengthens these findings. However, absolute risk increases were not always reported, which is crucial for clinical context.

Estrogen Dose Effect

A clear dose-response relationship was observed. Weill et al. (2016) found that lower estrogen doses were associated with lower risks of pulmonary embolism, with an HR of 1.9 (95% CI = 1.6-2.2) for 20 μg ethinylestradiol compared to 2.8 (95% CI = 2.4-3.3) for 30-40 μg [11].

Critical appraisal: This large cohort study (MMAT score 100%) provides robust evidence for the dose-effect relationship. However, confounding factors such as prescribing patterns for different risk groups could influence these results.

Progestogen Type Influence

Van Hylckama Vlieg et al. (2009) reported varying VTE risks by progestogen type, with ORs of 3.6 (95% CI = 2.9-4.6) for levonorgestrel, 5.6 (95% CI = 3.7-8.4) for desogestrel, and 7.3 (95% CI = 5.3-10.0) for drospirenone, all compared to non-users [33].

Critical appraisal: This case-control study (MMAT score 100%) provides detailed risk estimates by progestogen type. However, potential bias in prescribing patterns (e.g., newer progestogens prescribed to higher-risk women) should be considered.

Venous Versus Arterial Thrombosis

Peragallo Urrutia et al. (2013) found increased odds of venous thromboembolism (OR = 2.97, 95% CI = 2.46-3.59) and ischemic stroke (OR = 1.90, 95% CI = 1.24-2.91) with COC use, but not for myocardial infarction (OR = 1.34, 95% CI = 0.87-2.08) [34].

Critical appraisal: This systematic review and meta-analysis (MMAT score 100%) provides comprehensive risk estimates. The differing risks for venous and arterial events highlight the complex relationship between COCs and cardiovascular outcomes.

Duration of Use and Age Effects

Farley et al. (1998) reported that the risk of cardiovascular events increased with age and was highest in the first year of COC use [30]. However, specific risk ratios were not provided in the summary.

Critical appraisal: While this study provides important insights, its lower MMAT score (75%) and lack of specific risk estimates in the summary limit its impact. More recent, high-quality studies on duration effects would be valuable.

Progestin-Only Contraceptives

Tepper et al. (2016) found that most progestin-only contraceptives were not associated with increased thrombotic risk, except for some evidence of increased venous thromboembolism risk with injectables (specific risk estimates not provided in the summary) [9].

Critical appraisal: This systematic review (MMAT score 100%) offers important insights into the safety of progestin-only methods. However, the lack of specific risk estimates in the summary limits detailed interpretation.

Newer Formulations

Dinger et al. (2016) reported that a dienogest/estradiol valerate COC was associated with similar or lower cardiovascular risk compared to other COCs, although specific risk estimates were not provided in the summary [35].

Critical appraisal: This prospective cohort study (MMAT score 75%) suggests potential benefits of newer formulations. However, without specific risk estimates and given the potential for residual confounding, these results should be interpreted cautiously.

Risk After Discontinuation

Khader et al. (2003) found that past use of COCs was not significantly associated with increased myocardial infarction risk (OR = 1.15, 95% CI = 0.98-1.35) [31].

Critical appraisal: This meta-analysis (MMAT score 100%) provides reassuring data on risk reduction after COC discontinuation. However, the borderline confidence interval suggests that a small residual risk cannot be completely ruled out.

Overall, while the studies consistently demonstrate increased cardiovascular risks with COC use, the magnitude varies by formulation, user characteristics, and type of cardiovascular event. The highest quality evidence (systematic reviews and large cohort studies) provides the most reliable risk estimates. However, absolute risk increases, especially in young, healthy women, are generally low. Future research should focus on long-term effects, newer formulations, and strategies to minimize risk in vulnerable populations.

Discussion

Our review of 14 key studies reveals the complex relationship between hormonal contraceptives and cardiovascular risk. The evidence consistently shows an increased risk of venous thromboembolism associated with COCs, but the magnitude of this risk varies significantly across different formulations and populations.

Vinogradova et al. (2019) provided robust evidence from UK primary care databases, reporting adjusted ORs for venous thromboembolism ranging from 1.5 (95% CI = 1.3-1.7) for levonorgestrel-containing pills to 2.8 (95% CI = 2.4-3.3) for desogestrel-containing pills compared to non-use [37]. This gradient of risk across different progestogens was consistent with the meta-analysis by Stegeman et al. (2013), which found RRs of 3.6 (95% CI = 2.9-4.6) for levonorgestrel and 7.3 (95% CI = 5.3-10.0) for desogestrel compared to non-use [3].

However, the absolute risks remain low, particularly in young, healthy women. Lidegaard et al. (2011) reported incidence rates of venous thromboembolism per 10,000 woman-years ranging from 2.1 for non-users to 9.7 for users of third-generation pills [38]. This highlights the importance of considering baseline risk when interpreting RR increases.

The INAS-OC study by Dinger et al. (2016) challenged some of these findings, reporting no significant difference in venous thromboembolism risk between drospirenone-containing and other COCs (adjusted HR = 0.9, 95% CI = 0.5-1.6) [35]. This discrepancy underscores the need for cautious interpretation of observational data and highlights potential confounding factors in different study designs.

The impact of hormonal contraceptives on arterial thrombosis, including stroke and myocardial infarction, appears to be less pronounced than their effect on venous thrombosis. Chakhtoura et al. (2009) conducted a comprehensive review of the impact of combined hormonal contraceptives on metabolic parameters in women with and without metabolic disorders. They highlighted the complex interplay between hormonal contraceptives, lipid metabolism, and insulin resistance, factors that can influence long-term cardiovascular risk. However, the review did not provide quantitative risk estimates, limiting our ability to assess the magnitude of these effects [39].

Regarding arterial thrombosis, the meta-analysis by Roach et al. (2015) found a modestly increased risk of myocardial infarction (OR = 1.6, 95% CI = 1.2-2.1) and ischemic stroke (OR = 1.7, 95% CI = 1.5-1.9) among current COC users [12]. However, the absolute risks were very low, with excess incidence rates of two events per 10,000 woman-years for myocardial infarction and one event per 10,000 woman-years for ischemic stroke.

The impact of newer formulations on cardiovascular risk remains an area of active research. Dinger et al.’s (2016) INAS-SCORE study found no significant difference in cardiovascular events between a novel dienogest/estradiol valerate pill and established COCs (adjusted HR = 0.5, 95% CI = 0.2-1.3) [35]. However, the wide CI suggests that larger studies are needed to confirm these findings.

Stanczyk et al. (2013) reviewed the potential long-term clinical effects of oral contraceptives as postmenopausal hormone therapy, discussing both the potential risks and the possible cardioprotective effects in certain populations. They emphasized the need for longitudinal studies to fully elucidate the long-term cardiovascular impact of different contraceptive formulations [40].

The use of COCs in women with pre-existing cardiovascular risk factors requires careful consideration. Dragoman et al.’s (2018) systematic review found that while COCs can affect lipid profiles, the clinical significance of these changes remains uncertain [8]. They reported mean differences in lipid parameters that were statistically significant but of questionable clinical importance, such as a decrease in high-density lipoprotein cholesterol of 0.05 mmol/L (95% CI = -0.08 to -0.03) with levonorgestrel-containing COCs.

Tepper et al. (2016) conducted a systematic review of the safety of contraceptive use among women with medical conditions. They found that for many women with cardiovascular risk factors, the benefits of contraception outweigh the risks, but emphasized the need for individualized risk assessment. The impact of non-oral hormonal contraceptives on cardiovascular risk adds another layer of complexity. The systematic review by Tepper et al. (2016) suggested that the contraceptive patch may be associated with a higher venous thromboembolism risk than COCs (RR = 1.55, 95% CI = 1.10-2.19), while the vaginal ring showed no significant difference (RR = 1.11, 95% CI = 0.92-1.33) [9]. However, the quality of evidence was rated as low to moderate, highlighting the need for further research in this area.

The potential protective effects of hormonal contraceptives on certain aspects of cardiovascular health should also be considered. Shufelt and Bairey Merz (2009) discussed the potential antiatheromatous effects of contraceptive hormones, noting that some studies have suggested a possible reduction in atherosclerosis progression with certain formulations. However, they emphasized that these potential benefits must be weighed against the known thrombotic risks. The review did not provide quantitative measures of these potential protective effects, highlighting the need for further research in this area [18].

This potential protective effect was further explored by Samson et al. (2016), who conducted a retrospective cohort study examining cardiovascular disease incidence among females using different types of oral contraceptives. They found that some formulations as COC were associated with lower incidences of certain cardiovascular outcomes, suggesting potential cardioprotective effects. However, the study did not provide HRs or other quantitative measures of these effects, limiting our ability to assess their clinical significance [19].

The global perspective on hormonal contraceptive use and cardiovascular risk is crucial. Zakharova et al. (2015) conducted a study in Russia examining the cardiovascular risk profiles of women using different contraceptive methods. They found significant variations in risk factor prevalence and contraceptive choices compared to Western European and North American populations, highlighting the need for region-specific research and guidelines. However, the study did not provide comparative risk estimates, making it difficult to quantify these differences [13].

This global perspective was further emphasized by Plu-Bureau et al. (2013), who reviewed hormonal contraceptives and venous thromboembolism, focusing on differences in prescribing practices and risk profiles across different countries. The review highlighted the variability in venous thromboembolism risk estimates across studies and populations but did not provide pooled risk estimates, underscoring the complexity of generalizing findings across diverse populations [7].

Long-term cardiovascular implications of hormonal contraceptive use remain an area of uncertainty. Charlton et al.’s (2014) prospective cohort study using Nurses’ Health Study data found no significant increase in all-cause mortality (HR = 1.02, 95% CI = 0.99-1.04) or cardiovascular mortality (HR = 1.01, 95% CI = 0.96-1.08) among past users of oral contraceptives [17]. However, the study’s observational nature and potential for residual confounding limits the strength of these conclusions.

Similarly, Similarly, Angerer et al. (2001) conducted a trial, on oral postmenopausal hormone replacement on arterial diseases, including injectable and implantable methods. They found that these methods generally have a more favorable cardiovascular risk profile compared to combined hormonal contraceptives [41].

The interaction between hormonal contraceptives and other cardiovascular risk factors is crucial for clinical decision-making. Horton et al.’s (2016) systematic review found that obesity significantly increased the already elevated venous thromboembolism risk associated with COCs, with obese COC users having a 5-8-fold higher risk compared to normal-weight non-users [42]. This underscores the importance of considering multiple risk factors in contraceptive counseling.

In conclusion, while the overall body of evidence supports an increased cardiovascular risk with hormonal contraceptives, particularly COCs, the absolute risks remain low for most women. The variation in risk across different formulations and populations highlights the need for personalized risk assessment and shared decision-making in contraceptive choice. Future research should focus on long-term cardiovascular outcomes, the impact of newer formulations, and strategies for optimizing the risk-benefit balance in high-risk populations.

## Conclusions

This systematic review demonstrates that while hormonal contraceptives are associated with an increased risk of cardiovascular events, particularly venous thromboembolism, the absolute risk for most women remains low. The risk varies with the type and dose of hormonal components and is influenced by individual risk factors. Lower estrogen doses and certain progestogens appear to carry lower risks, and progestin-only methods generally have a favorable cardiovascular risk profile. These findings underscore the importance of individualized assessment and counseling when prescribing hormonal contraceptives. Healthcare providers should consider a woman’s age, personal and family medical history, and lifestyle factors when discussing contraceptive options. The benefits of effective contraception should be weighed against the potential cardiovascular risks, taking into account individual preferences and circumstances.
